# Budesonide and Formoterol Reduce Early Innate Anti-Viral Immune Responses *In Vitro*

**DOI:** 10.1371/journal.pone.0027898

**Published:** 2011-11-18

**Authors:** Janet M. Davies, Melanie L. Carroll, Hongzhuo Li, Alisa M. Poh, Darren Kirkegard, Michelle Towers, John W. Upham

**Affiliations:** 1 Lung and Allergy Research Centre, School of Medicine, The University of Queensland, Princess Alexandra Hospital Clinical Division, Woolloongabba, Queensland, Australia; 2 Department of Respiratory Medicine, Princess Alexandra Hospital, Woolloongabba, Queensland, Australia; Centre de Recherche Public de la Santé (CRP-Santé), Luxembourg

## Abstract

Asthma is a chronic inflammatory airways disease in which respiratory viral infections frequently trigger exacerbations. Current treatment of asthma with combinations of inhaled corticosteroids and long acting beta2 agonists improves asthma control and reduces exacerbations but what impact this might have on innate anti-viral immunity is unclear. We investigated the *in vitro* effects of asthma drugs on innate anti-viral immunity. Peripheral blood mononuclear cells (PBMC) from healthy and asthmatic donors were cultured for 24 hours with the Toll-like receptor 7 agonist, imiquimod, or rhinovirus 16 (RV16) in the presence of budesonide and/or formoterol. Production of proinflammatory cytokines and expression of anti-viral intracellular signalling molecules were measured by ELISA and RT-PCR respectively. In PBMC from healthy donors, budesonide alone inhibited IP-10 and IL-6 production induced by imiquimod in a concentration-dependent manner and the degree of inhibition was amplified when budesonide and formoterol were used in combination. Formoterol alone had little effect on these parameters, except at high concentrations (10^−6^ M) when IL-6 production increased. In RV16 stimulated PBMC, the combination of budesonide and formoterol inhibited IFNα and IP-10 production in asthmatic as well as healthy donors. Combination of budesonide and formoterol also inhibited RV16-stimulated expression of the type I IFN induced genes myxovirus protein A and 2′, 5′ oligoadenylate synthetise. Notably, RV16 stimulated lower levels of type Myxovirus A and oligoadenylate synthase in PBMC of asthmatics than control donors. These *in vitro* studies demonstrate that combinations of drugs commonly used in asthma therapy inhibit both early pro-inflammatory cytokines and key aspects of the type I IFN pathway. These findings suggest that budesonide and formoterol curtail excessive inflammation induced by rhinovirus infections in patients with asthma, but whether this inhibits viral clearance *in vivo* remains to be determined.

## Introduction

Asthma is a chronic inflammatory disease of the lower airways affecting up to 300 million individuals worldwide and posing a significant burden on health care systems in both western and developing countries [1]. Asthma adversely affects patient quality of life, productivity and absenteeism from school or work [2]. However, the major medical burden and health care costs of asthma including morbidity and mortality occur during acute exacerbations [3], [4]. Up to 80% of episodes of acute asthma in children and 70% in adults are attributed to respiratory viral infection with rhinovirus being a major culprit [5], [6], [7], [8].

Current asthma treatment usually includes a combination of inhaled corticosteroids and long acting beta2 agonists. These medications are effective at controlling symptoms of asthma and they reduce but do not eliminate asthma exacerbations [9], [10], [11]. Moreover, the effects of these medications on host defence against virus infections remains unclear. Induction of innate anti-viral immunity is necessary for initiation for viral clearance and recruitment of virus-specific adaptive immune responses [12], [13], [14]. However, the excessive pro-inflammatory responses in asthmatic individuals could contribute to the immunopathology of acute episodes of asthma [15].

Initiation of immune responses against respiratory viral infection involves both structural cells of the lungs and leukocytes recruited from the circulation [16]. Although the respiratory mucosa is the primary site of rhinoviral infection, migratory cells originating in the bone marrow are likely to make a major contribution to host defence against this virus [17]. There is increasing evidence that asthma is associated with changes in the anti-viral function of blood leukocytes [18], [19], [20], [21]. Sampling the peripheral blood provides a way to ‘intercept and interrogate’ a variety of immune cells which are *en route* to the lungs and regional lymph nodes. Furthermore, experimental rhinovirus infections in asthmatics have shown a strong correlation between *in vitro* responses of peripheral blood leukocytes to rhinovirus and clinically relevant *in vivo* outcomes including asthma symptoms, bronchial hyper-responsiveness and the extent of viral shedding [22], [23], [24]. Whilst the effect of corticosteroids and long acting beta2 agonists on rhinovirus infected bronchial epithelial cells have been investigated previously [25], [26], [27], there is little information regarding the effects of these treatments on early innate immune responses of peripheral blood leukocytes.

In this study we aimed to investigate the effect of combinations of the glucocorticoid, budesonide, and the long acting beta2 agonist, formoterol, on innate immune responses to rhinovirus. Rhinoviruses are single stranded RNA viruses that are likely to have complex effects on host cells including interactions with host defence molecules that detect viral nucleic acids. We have previously shown that adolescents with asthma have a reduced responses of peripheral blood leukocytes to agonists for the Toll-like receptor for single stranded RNA (TLR7) [21]. Therefore we first examined peripheral blood mononuclear cells that were activated via TLR7 before conducting a series of experiments with live rhinovirus strain 16 (RV16). Because differences in innate interferon responses to rhinovirus between healthy and asthmatic donors have been reported elsewhere [19], [20], [28], we examined the *in vitro* effects of budesonide and formoterol on various aspects of innate host immunity in healthy as well as asthmatic donors.

## Results

### Subject Characteristics

We previously observed differences in adaptive immunity to rhinovirus between healthy premenopausal women and aged-matched men [31]. However, innate immunity to rhinovirus was not affected by age or sex in that study. Nonetheless, to eliminate any potential gender-based confounding effects for the experiments with RV16, 11 of 12 healthy donors and all asthmatic PBMC donors were female. Healthy control and asthmatic donors had similar body mass index (Table 1). The healthy donors were slightly but significantly older than the asthmatic donors, but this is unlikely to have a biological effect since most participants in both groups were premenopausal in age. Asthmatic donors had mild to moderate doctor-diagnosed asthma. Six of twelve asthmatics were prescribed inhaled corticosteroids (budesonide or fluticasone propionate in combination with formoterol or salmeterol) and one was prescribed a nasal corticosteroid spray but none were taking oral steroids. The asthmatic donors were all allergic to house dust mites with or without allergic sensitivities to other aeroallergens whereas the control group were all non-atopic (Table 1).

**Table 1:** Patient characteristics of PBMC donors for experiments comparing the effects of asthma drugs on innate immunity to RV16.

Variable	Asthmatic donors	Healthy donors
Number	12	12
Female	12	11
Age (median and IQ range)	30.00 (28.33–40.91) *	43.46 (33.54–50.81)
Body mass index (median and IQ range)	25.25 (21.50–27.08)	23.65 (21.01–30.53)
Sum skin prick diameters for 10 common aeroallergens (median and IQ range)	16.5 (10.25–37.1)**	0 (0–0)

*Difference between asthmatic and healthy donors by Mann Whitney U test (p = 0.0106).

**Difference between asthmatic and healthy donors by Mann Whitney U test (p<0.0001). The most frequently recognised allergens were house dust mite (n = 12), grass pollen (n = 6) cat dander (n = 6) and Alternaria (n = 4).

### Effects of budesonide and formoterol on TLR7 activated PBMC from healthy donors

**Figure 1:**
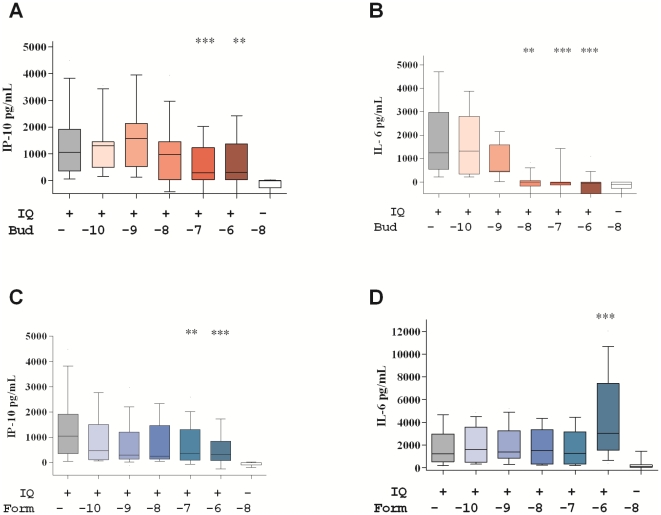
Dose dependent effects of budesonide and formoterol on markers of innate immunity induced by imiquimod (IQ). IP-10 (A, C) and IL-6 (B, D) production by PBMC from 20 healthy female donors cultured with IQ in the presence of increasing concentrations (10^−10^–10^−6^ M as indicated) of budesonide (Bud) and formoterol (Form). The background cytokine levels produced in unstimulated control cultures (median 69 pg/ml IP-10 and 181 pg/ml IL-6) have been subtracted. Box and whisker plots show the median and interquartile range with 10^th^–90^th^ percentile. Differences between IQ-stimulated cultures and IQ-stimulated cultures with drugs were tested by Friedman ANOVA and Dunn's multiple comparison test (**, p<0.005; ***, p<0.001).

The initial experiments focused on TLR7-activated PBMC. Cells from healthy donors were stimulated with the TLR7 agonist IQ in the presence of increasing concentrations of budesonide and formoterol. PBMC cultures stimulated with IQ produced large amounts of the type I IFN-associated chemokine IP-10. Treatment with high concentrations of budesonide (10^−7^–10^−6^ M) significantly reduced IQ-induced IP-10 production with a maximum of 80% inhibition at 10^−6^ M budesonide (Figure 1A). Treatment with budesonide alone had a stronger effect on the pro-inflammatory cytokine IL-6 production with a concentration-dependent reduction and reaching complete inhibition of IL-6 at 10^−8^ M budesonide (Figure 1B).

Formoterol also diminished IP-10 production induced by IQ with significant inhibition of 76% observed at 10^−6^ M formoterol (Figure 1C). In contrast, formoterol did not reduce IL-6 production stimulated by IQ (Figure 1D). In fact, there was a 2.8 fold enhancement of IL-6 production induced by IQ at 10^−6^ M formoterol, suggesting independent regulation of IP-10 and IL-6.

**Figure 2:**
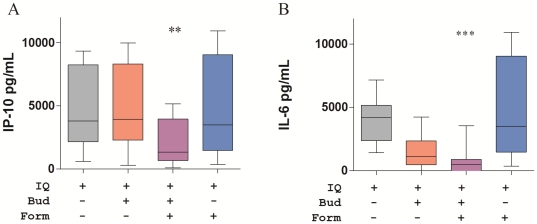
Effect of combination of budesonide and formoterol on imiquimod (IQ)-induced markers of innate immunity. IP-10 (A) and IL-6 (B) production by PBMC from eight healthy female donors were cultured with IQ with budesonide (Bud, 10^−8^ M) and/or formoterol (Form,10^−8^ M). The background cytokine levels produced in unstimulated control cultures (median 211 pg/ml IP-10 and 238 pg/ml IL-6) have been subtracted. Box and whisker plots show the median and interquartile range with 10^th^–90^th^ percentile. Differences between IQ-stimulated cultures and IQ-stimulated cultures with drugs were tested by Friedman ANOVA and Dunn's multiple comparison test (**, p<0.005; ***, p<0.001).

We next investigated the effects of combinations of budesonide and formoterol at pharmacologically relevant concentrations. The combination of budesonide and formoterol (10^−8^ M of each drug) inhibited IQ-stimulated IP-10 production, whereas when tested individually at the same concentrations, these drugs showed no effect (Figure 2A). The reduction in IQ-stimulated IL-6 production observed with budesonide treatment was further enhanced by addition of formoterol (Figure 2B).

### Effects of budesonide and formoterol on IFNα production by RV16-stimulated PBMC from healthy and asthmatic donors

**Figure 3:**
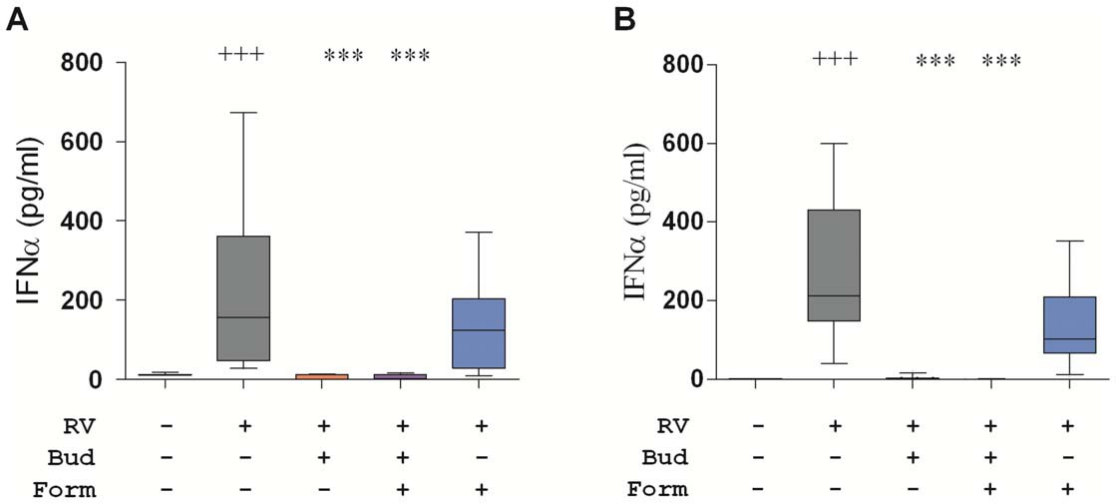
Effects of combination of budesonide and formoterol on IFNα-induced by rhinovirus. Cytokines produced by PBMC from healthy (A) and asthmatic (B) donors cultured for 24 h with RV16 in the presence of budesonide (Bud, 10^−8^ M) and/or formoterol (Form,10^−8^ M) as indicated. Box and whisker plots show median, interquartile range, and 10^th^ and 90^th^ percentiles for data from 12 donors in each subject group. Significant differences by Friedman ANOVA and Dunn's multiple comparison test for unstimulated versus RV16-stimulated cultures (+++, p<0.005) and between RV16-stimulated and RV16-stimulated cultures treated with budesonide and/or formoterol (***, p<0.001).

Having shown that combinations of budesonide and formoterol reduced TLR7-induced cytokine production, the following series of experiments examined the effects of these drugs on RV16-stimulated PBMC. RV16 induced IFNα production to a similar extent in cells from both healthy and asthmatic donors (Figure 3A, B). Budesonide (10^−8^ M) alone and in the combination with formoterol (10^−8^ M) completely blocked IFNα production induced by RV16, so it was impossible to determine if the combination with formoterol had additional effects on IFNα production. The degree of inhibition of IFNα achieved with budesonide alone or in combination with formoterol was similar in both healthy and asthmatic donors. Although formoterol alone appeared to induce a modest reduction in RV16-stimulated IFNα in PBMC from both healthy and asthmatic donors, this reduction was not statistically significant.

### Effects of budesonide and formoterol on molecules downstream of IFNα

**Figure 4:**
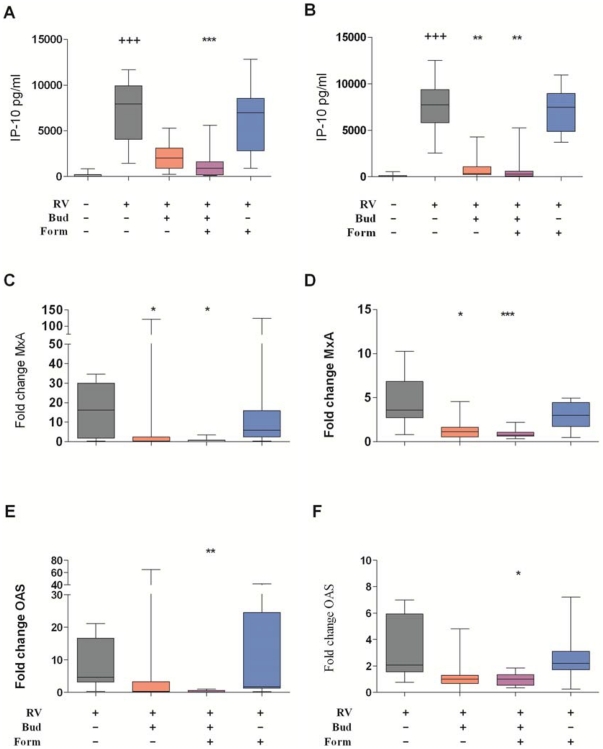
Effect of budesonide and formoterol on rhinovirus-induced expression of type one IFN inducible genes. IP-10 produced by PBMC from healthy (A) and asthmatic (B) donors cultured for 24 h with RV16 in the presence of budesonide (Bud, 10^−8^ M) and/or formoterol (Form, 10^−8^ M) as indicated. Box and whisker plots show median, interquartile range, 10^th^ and 90^th^ percentiles for data. Quantitative RT-PCR analysis of anti-viral genes MxA and OAS expression in healthy donors (C and E, n = 8) and asthmatic donors (D and F, n = 12). Significant differences by Friedman ANOVA and Dunn's multiple comparison test for unstimulated versus RV16-stimulated cultures (+++, p<0.005) and between RV16 versus RV16 with drugs were assessed by Friedman ANOVA and Dunn's multiple comparison test (*, p<0.05; **, p<0.005, ***, p<0.001). To show the effects of the drugs on gene expression the data for asthmatics is shown on a different scale to that from healthy donors.

RV16 strongly induced production of similarly high levels of the type I interferon responsive chemokine IP-10 in PBMC cultures of healthy and asthmatic donors. In healthy donors, treatment with budesonide (10^−8^ M) reduced RV16-induced IP-10 production by 78.1% (Figure 4A), whereas 10^−9^ M budesonide alone showed no effect (Supplementary Figure S1A). This IP-10 production was further reduced by addition of formoterol (10^−8^ M, Figure 4A), although formoterol alone had no effect at this or higher concentration (10^−8^ M, Figure 4A and 10^−7^ M, data not shown).

Budesonide (10^−8^ M) inhibited RV16-induced IP-10 to a significantly greater extent in cultures from asthmatic individuals than in cultures from healthy individuals (p = 0.022). In fact the degree of inhibition of IP-10 induced by budesonide in the asthmatics was so complete that it was not possible to determine if formoterol had additional inhibitory effects (Figure 4B). However, with lower concentrations of budesonide (10^−9^ M), combination with formoterol (10^−8^ M) further inhibited IP-10 synthesis by RV16- stimulated PBMC from asthmatic donors (supplementary Fig. S1B). Formoterol alone had no effect on RV-induced IP-10 (10^−8^ M, Figure 4B, and 10^−7^ M, data not shown).

MxA and OAS are two important anti-viral molecules downstream of type I IFN. RV16 strongly induced mRNA expression of both MxA and OAS in cultures of PBMC from all healthy donors (Figure 4 C, E). In healthy donors, budesonide alone, but not formoterol, inhibited rhinovirus induction of MxA whereas neither budesonide nor formoterol alone reduced rhinovirus-induced expression of OAS. However, there was nearly complete inhibition of MxA (95.8%) and OAS expression (96.1%) by the combination of budesonide and formoterol.

In PBMC from asthmatic donors, RV16 also induced expression of type I IFN stimulated genes (Figure 4 D, F), though the magnitude of MxA and OAS expression was significantly less than in PBMC from healthy donors (Table 2). Budesonide treatment of PBMC from asthmatic donors significantly reduced RV16 induction of MxA expression (Figure 4D). The apparent reduction in RV16-induced OAS by budesonide was not statistically significant (Figure 4F). The combination of budesonide and formoterol inhibited MxA and OAS induction by RV16 in PBMC cultures of asthmatic donors by 78.8% and 73.5%, respectively, and this was significantly lower degree of inhibition than in PBMC from healthy donors (p = 0.007 for MxA and p = 0.0008 for OAS).

**Table 2:** Comparison of response to rhinovirus 16 between healthy and asthmatic donors.

Parameter	Healthy donors	Asthmatic donors
IFNα (pg/ml)	155 (47–360)	213 (149–430)
IP-10 (pg/ml)	7956 (4056–9920)	7729 (5806–9375)
IL-6 (pg/ml)	297 (150–412)	295 (150–1702)
MxA (cDNA fold induction)	16.2 (1.8–30.1)	3.85 (2.7–6.8)*
OAS (cDNA fold induction)	4.6 (3.1–16.7)	2.1 (1.5–5.9)**

Data is presented as median (interquartile range).

*MxA expression was significantly lower in asthmatic donors p = 0.0182.

*OAS expression was significantly lower in asthmatic donors p = 0.039.

### Effects of budesonide and formoterol on other innate cytokines induced by RV16 in PBMC from healthy and asthmatic donors

RV16 stimulation did not significantly induce IL-6 production above that seen in unstimulated PBMC cultures (Supplementary Figure S2). Nonetheless, the small amount of IL-6 produced was completely abolished by treatment with budesonide (10^−8^ M) in both healthy and asthmatic donors such that further effects of formoterol (10^−8^ M) addition were not observed (Supplementary Figure S2). At 10^−9^ M, budesonide did not significantly inhibit IL-6 production with or without addition of formoterol (10^−7^ or 10^−8^ M) (data not shown). Formoterol alone at these concentrations had no significant effect on RV-induced IL-6 production in healthy or asthmatic donor PBMC (Supplementary Figure S2, and data not shown).

Significant amounts of IL-8 and TNFα were not detected in PBMC cultures exposed to RV16 for 24 h from either healthy or asthmatic donors (data not shown).

## Discussion

Rhinoviruses are common triggers for acute exacerbations of asthma and are a significant cause of asthma morbidity and mortality [3]. Inhaled corticosteroids and long acting beta2 agonists form the cornerstones of current asthma therapy [9], [33], and even in previously healthy individuals these agents are sometimes used in an effort to reduce persistent airway symptoms such as prolonged coughing after respiratory viral infections. Combination therapy with corticosteroids and long acting beta2 agonists affords the advantage of limiting the dose of corticosteroids required to treat asthma [33]. However, it is not clear whether corticosteroids and long acting beta2 agonists alter the capacity of circulating immune cells to mount an early innate immune response to rhinovirus.

Here we observed that budesonide inhibited TLR7-induced synthesis of IP-10 and IL-6 in PBMC, and that the degree of inhibition was usually enhanced when budesonide was used in combination with formoterol. Similarly, in RV16 stimulated PBMC budesonide inhibited various aspects of innate anti-viral responses, and mostly the degree of inhibition was enhanced when budesonide was used in combination with formoterol. The inhibitory effects of these drugs were seen in cultures of PBMC from healthy as well as asthmatic donors.

Our observation that combination of budesonide and formoterol treatment inhibited early innate anti-viral immunity in circulating leukocytes is consistent with previous research using structural cells of the lung. In these studies budesonide and formoterol or fluticasone propionate and salmeterol inhibited IP-10 and IL-8 production induced by rhinovirus in cultured bronchial epithelial cell lines and primary human epithelial cells from healthy subjects [25], [34]. Glucocorticoids have also been shown to block rhinovirus-induced IP-10 production in airway fibroblasts (Thomas and Bardin, personal communication). In cultured airway smooth muscle, airway epithelial cells and lung fibroblasts, combinations of these drugs exhibited synergistic reduction in pro-inflammatory responses, chemokines and extracellular matrix molecules involved in airway remodeling [25], [27], [34], [35], [36], [37].

Interestingly, we observed an increase in IQ-induced IL-6 production in the presence of extra high dose formoterol (10^−6^ M; see Figure 1D). This finding is consistent with observations that RV16-induced IL-6 production in the bronchial epithelial cell line BEAS-2B was increased 2–3 fold by salmeterol (10^−9^ M and 10^−8^ M) [26], and that RV1b induction of IL-6 in bronchial epithelial cell tended to increase by 40–50% at high doses of formoterol (10^−7^ M and 10^−6^ M) [25]. These findings suggest that use of salmeterol and formoterol in the absence of corticosteroids may have pro-inflammatory effects. Also, the T helper 2 cell promoting factor macrophage derived chemokine was enhanced in bronchial epithelial cells in response to formoterol and salmeterol suggesting that long acting beta2 agonists by themselves could favour Th2 inflammation in the lung [38].

The contribution of rhinoviruses to acute exacerbations of asthma has been well documented [5], [6], [7], [8] but debate continues as to whether the effects are directly due to the virus itself or indirectly due to an aberrant host response to the virus. Following experimental rhinovirus infection Message and colleagues reported that the severity of lower airway symptoms was associated with viral load [23]. In contrast, a longitudinal study of natural rhinoviral infections in asthmatics indicated that severity of lower respiratory tract symptoms was not related to viral load [39]. A prospective study of cohabiting couples in UK (where one person had atopic asthma and one was healthy) showed that whilst asthmatics did not differ from healthy individuals in the frequency of rhinovirus infections, the duration and severity of the infections were increased in those with asthma [40]. Collectively, these studies suggest that an aberrant immune response to rhinovirus contributes to the induction and severity of symptoms following rhinovirus infection in people with asthma, though how much of this is due to asthma *per se*, and how much can be attributed to medications used to treat asthma has not been investigated in detail. Grunberg et al. found that pre-treatment with budesonide decreased eosinophilic inflammation at six days post RV16 challenge but overall the mild airway inflammation induced by RV16 was not affected, for better or worse, by budesonide [41]. However, in that study combination therapy with inhaled steroids and long acting beta2 agonists was not examined, nor was there any examination of early innate anti-viral immunity or viral clearance [41].

Even though the combination of budesonide and formoterol clearly inhibit aspects of anti-viral immunity, especially the type I IFN pathway, the implications this has for airway inflammation and host defence are not clear. Reduction of chemokines such as IP-10 production would be expected to diminish recruitment of activated T lymphocytes, key protagonists for immune responses to rhinovirus. Reduced production of IFNβ by bronchial epithelial cells in asthma is thought to prevent effective anti-viral responses and delay viral clearance [42], [43]. Furthermore, PBMC from asthmatics synthesise less IFNα2 when infected with respiratory syncytial virus or Newcastle disease virus [20]. However, excessive IP-10, a cytokine downstream of type I IFN, produced in response to rhinovirus was associated with increased risk of asthma [42].

The primary aim of this study was to investigate the effects of common asthma drugs on anti-viral innate immunity rather than differences between healthy and asthmatic donors. Nonetheless, while there were no detectible differences between healthy and asthmatics donors in respect to IFNα production, RV16 induced significantly lower expression of two important anti-viral molecules, MxA and OAS, in asthmatics compared with healthy subjects. This data is consistent with the notion that innate anti-viral immunity is impaired in asthmatics [43], [44]. Together it appears that in asthma the dysregulated immunity to respiratory viral infections is mediated not only by structural cells in the lung but also by circulating leukocytes, possibly pDC [45], [46].

The clinical consequences of the effects of budesonide and formoterol on anti-viral immunity remain to be established. Inhibition of inflammatory cytokine production and airway inflammation is clearly a worthwhile therapeutic aim in asthma, and there is compelling clinical evidence that these drugs are beneficial in asthma. However, there remains a degree of concern that inhibition of already impaired type I IFN might lead to delayed viral clearance in some people with asthma. Some investigators are already examining whether administration of type I IFN might be beneficial in asthma. In a small study, 10 severe steroid resistant asthmatics were afforded amelioration of their asthma with intravenous low dose IFNα treatment suggesting a protective role for type I IFN [47]. Moreover, type I IFN treatment may provide benefits for patients with acute exacerbations of asthma triggered by rhinoviral infection [48].

Budesonide and formoterol are capable of down regulating both pro-inflammatory and type 1 IFN responses of peripheral blood leukocytes stimulated with rhinovirus *in vitro.* Given the importance of the type I IFN response in protection against rhinovirus in healthy and asthmatic donors and the complex interactions between migratory immune cells derived from the circulation and structural cells of the respiratory tract, there is now a clear need for further *in vivo* studies examining the effects of budesonide and formoterol therapy upon viral clearance and duration of symptoms following rhinovirus challenge in patients with asthma.

## Methods

### Study participants

Volunteers were recruited from the Respiratory Clinic, Princess Alexandra Hospital and from normal healthy laboratory and healthcare workers. The subjects of the study were skin prick tested to a panel of ten common areoallergen extracts including house dust mite, grass and ragweed pollens, molds and cat dander (Holister Stier, Spokane, WA, USA). Participants completed a detailed questionnaire documenting symptoms of lung disease and atopic disorders such as allergic rhinitis and eczema, and current medication use. All asthmatic subjects had a prior diagnosis of asthma confirmed by a doctor and a history of typical mild to moderate asthma symptoms within the last 12 months. All healthy control subjects were free of respiratory symptoms and had negative allergen skin prick tests. The study was conducted in accordance to the Declaration of Helsinki and was approved by the Princess Alexandra Hospital and the University of Queensland Human Research Ethics Committees. Informed written consent was obtained from each subject.

Peripheral blood mononuclear cells were isolated from heparinised blood using Lymphoprep (Axis-shield, Oslo, Norway) and cryopreserved for subsequent experiments as previously described [21].

### Culture conditions

Ohio HeLa cells and the major group rhinovirus strain RV16 were kindly donated by Professor Phil Bardin (Monash Medical Centre, Melbourne, Australia). RV16 was propagated in Ohio HeLa cells and purified over a sucrose gradient as previously described [29], [30]. Imiquimod (IQ, Invivogen, San Diego, CA, USA), formoterol and budesonide (kindly donated by Astra Zeneca, Macclesfield Cheshire SK10NA, United Kingdom) were dissolved in dimethylsulfoxide at 10^−2^ M and frozen in aliquots until use. The final concentration of DMSO in the cultures was negligible; at the highest concentration of budesonide and formoterol at 10^−6^ M, there was 0.001% v/v DMSO). Duplicate wells with 2.5×10^5^ PBMC per well at a density of 1×10^6^ per millilitre were cultured in RPMI media supplemented with penicillin, streptomycin, glutamate, 2-mercaptoethanol and 10% foetal calf serum. Cells were cultured with and without IQ (1 µg/ml) or rhinovirus 16 (RV16) at a multiplicity of infection of one. To replicate cultures with IQ or RV16 various combinations of budesonide and formoterol from 10^−10^ M to 10^−6^ M were added. Cultures were incubated at 37°C with 5% CO_2_. Cell pellets from cultures harvested at 6 hour were stored in RNA-protect (Qiagen, Hilden, Germany) until RNA was extracted. Supernatants from cells cultured for 24 hour were harvested for cytokine quantification by ELISA.

### ELISA

IP-10 (CXCL10), IL-6, TNFα and IL-8 ELISAs were performed using commercially available paired antibodies and recombinant cytokines (Becton Dickenson, Franklin Lakes, NJ, USA) and IFNα was assayed via ELISA kit (PBL Interferon Source, Piscataway, NJ, USA) according to the manufacturer's instructions. The lower limits of detection of these assays were as follows: IP10, 3.9 pg/ml; IL-6 and TNFα, 4.0 pg/ml; IFNα,4.9 and IL-8 7.8 pg/ml.

#### Quantitative Real Time PCR

RNA was extracted using RNeasy or RNeasy plus Spin kit and reverse transcribed using Quantitect reverse transcription kit (Qiagen), according to manufacturer's instructions. Real Time (RT-) PCR was performed with Quantitect SYBR green PCR mix (Qiagen). The expression of mRNA transcripts for Myxovirus protein A (MxA) and 2′,5′-oligoadenylate sythetase (OAS) were determined taking into account the efficiency of amplification of values normalized to expression of the reference Ubiquitin containing enzyme D2 gene as described [31], [32].

### Statistics

Statistical analysis was performed using SPSS 18 (IBM SPSS Inc., Chicago, IL, USA). Data was assessed for normality by Kolmogorov-Smirnov test. Differences between IQ or RV16 stimulated cultures with and without drugs were analysed by non-parametric Friedman ANOVA with Dunn's pairwise multiple comparison tests, with p<0.05 considered significant. When values for responses to stimuli were normally distributed then differences between healthy and asthmatic donors were assessed by a Student's T test.

## Supporting Information

Figure S1 Effects of other concentrations of combined budesonide and formoterol on IP-10 induced by rhinovirus. Cytokines produced by PBMC from age- and body mass index-matched female healthy (A) and asthmatic (B) donors cultured for 24 h with RV16 in the presence of budesonide (Bud, 10^−9^ M) and/or formoterol (Form,10^−8^ M) as indicated. Box and whisker plots show median, interquartile range, and 10^th^ and 90^th^ percentiles for data from 12 donors in each subject group. Significant differences by Friedman ANOVA and Dunn's multiple comparison test for unstimulated versus RV16-stimulated cultures (++, p<0.005) and for RV16 versus RV16 with drugs (*, p<0.05).
(TIF)

Figure S2 Effects of combination of budesonide and formoterol on IL-6 induced by rhinovirus. Cytokines produced by PBMC from age- and body mass index-matched female healthy (A) and asthmatic (B) donors cultured for 24 h with RV16 in the presence of budesonide (Bud, 10^−8^ M) and/or formoterol (Form, 10^−8^ M) as indicated. Data shown as median, interquartile range and 10^th^ and 90^th^ percentiles for data from 12 donors. Significant differences by Friedman ANOVA and Dunn's multiple comparison test for RV-stimulated cultures versus RV-stimulated cultures with drugs (*, p<0.05; ***, p<0.001).
(TIF)
