# Supplementary figures and images for: Budesonide and Formoterol Reduce Early Innate Anti-Viral Immune Responses In Vitro
Source: PLoS One. 2011 Nov 18;6(11):e27898. doi: 10.1371/journal.pone.0027898 (PMC3220700; doi:10.1371/journal.pone.0027898)

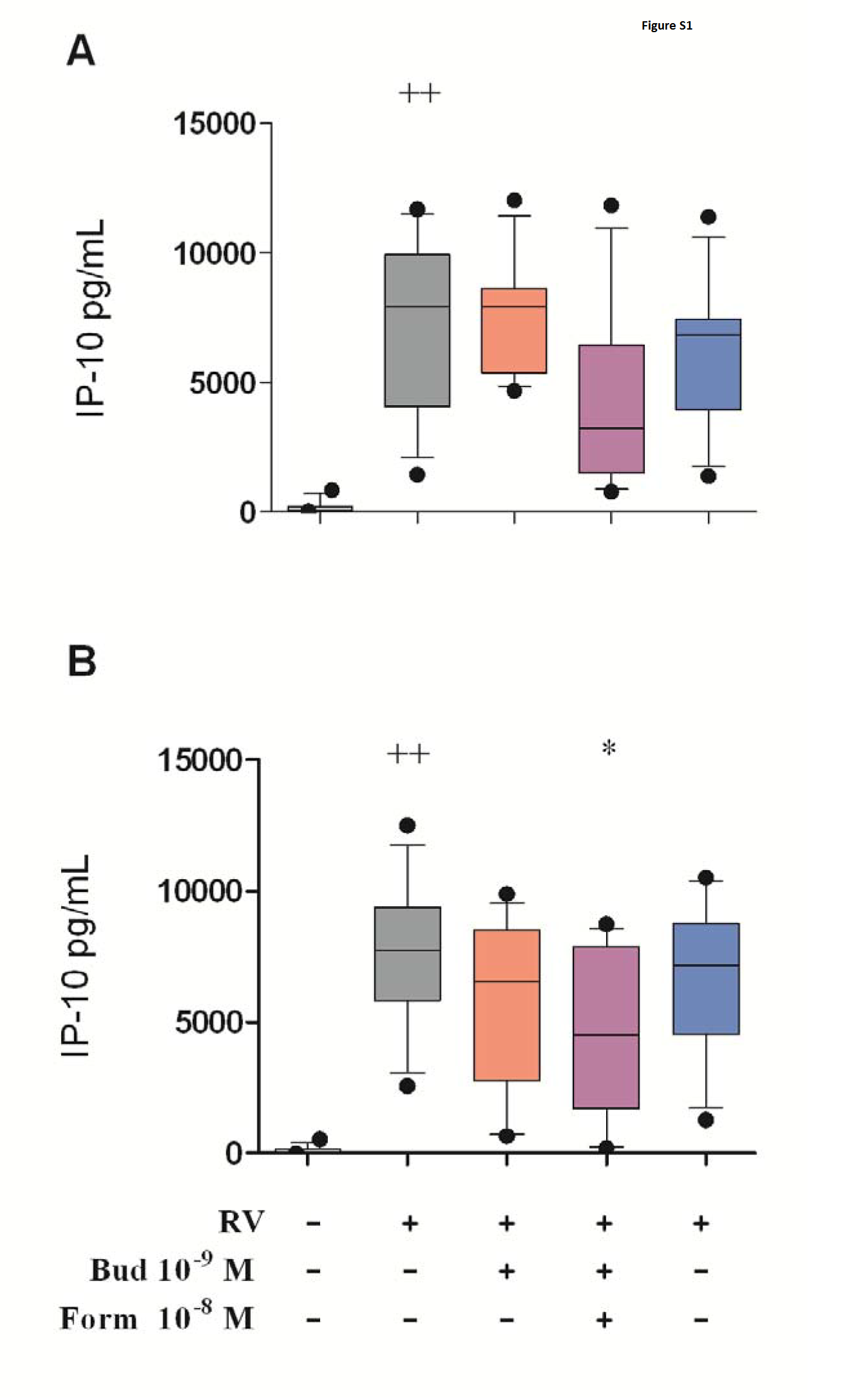

Supplement: Figure S1 — Effects of other concentrations of combined budesonide and formoterol on IP-10 induced by rhinovirus. Cytokines produced by PBMC from age- and body mass index-matched female healthy (A) and asthmatic (B) donors cultured for 24 h with RV16 in the presence of budesonide (Bud, 10−9 M) and/or formoterol (Form,10−8 M) as indicated. Box and whisker plots show median, interquartile range, and 10th and 90th percentiles for data from 12 donors in each subject group. Significant differences by Friedman ANOVA and Dunn's multiple comparison test for unstimulated versus RV16-stimulated cultures (++, p<0.005) and for RV16 versus RV16 with drugs (*, p<0.05). (TIF) [file pone.0027898.s001.tif]

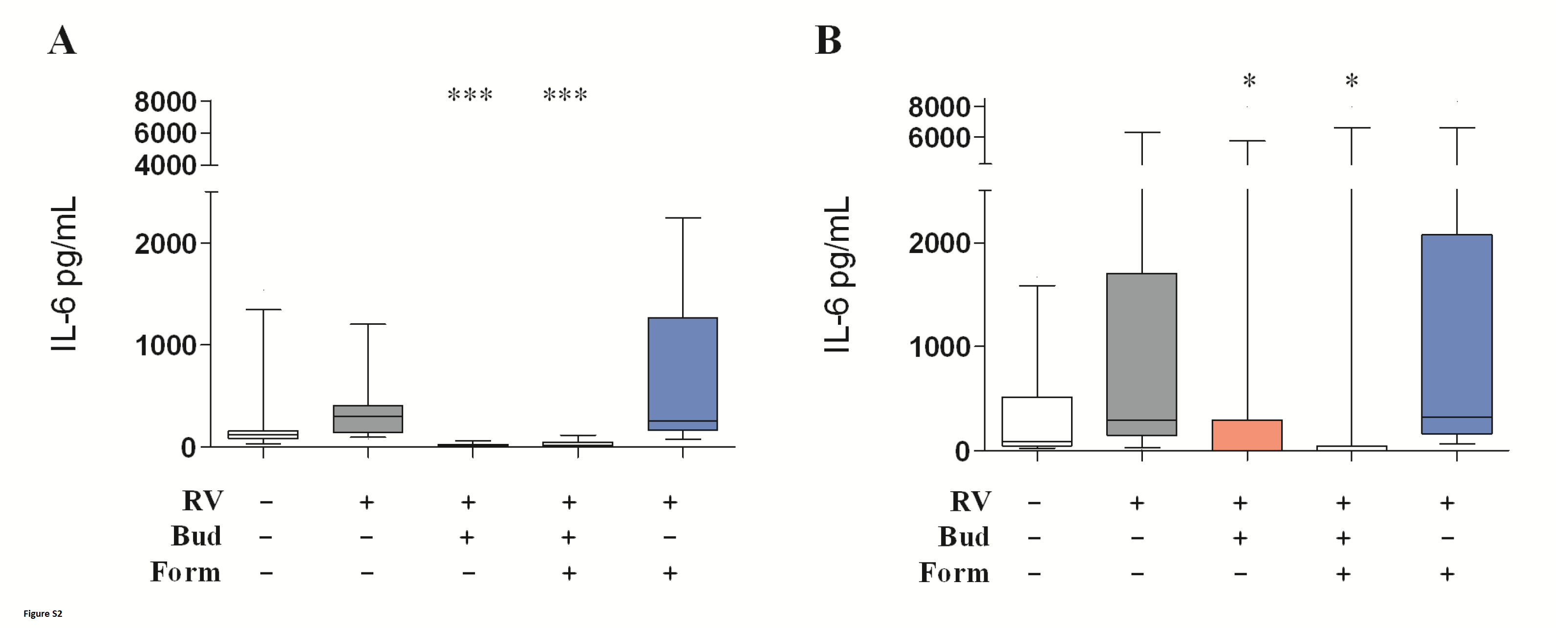

Supplement: Figure S2 — Effects of combination of budesonide and formoterol on IL-6 induced by rhinovirus. Cytokines produced by PBMC from age- and body mass index-matched female healthy (A) and asthmatic (B) donors cultured for 24 h with RV16 in the presence of budesonide (Bud, 10−8 M) and/or formoterol (Form, 10−8 M) as indicated. Data shown as median, interquartile range and 10th and 90th percentiles for data from 12 donors. Significant differences by Friedman ANOVA and Dunn's multiple comparison test for RV-stimulated cultures versus RV-stimulated cultures with drugs (*, p<0.05; ***, p<0.001). (TIF) [file pone.0027898.s002.tif]
